# *CHD8* Variant and Rett Syndrome: Overlapping Phenotypes, Molecular Convergence, and Expanding the Genetic Spectrum

**DOI:** 10.1155/humu/5485987

**Published:** 2025-06-02

**Authors:** Elaine Zhang, Teresa Zhao, Tim Sikora, Carolyn Ellaway, Wendy A. Gold, Nicole J. Van Bergen, David A. Stroud, John Christodoulou, Simranpreet Kaur

**Affiliations:** ^1^Department of Paediatrics, University of Melbourne, Melbourne, Victoria, Australia; ^2^Murdoch Children's Research Institute, Royal Children's Hospital, Melbourne, Victoria, Australia; ^3^Victorian Clinical Genetics Services, Royal Children's Hospital, Melbourne, Victoria, Australia; ^4^Genetic Metabolic Service, The Children's Hospital at Westmead, Sydney Children's Hospital Network, Sydney, New South Wales, Australia; ^5^Faculty of Medicine and Health, University of Sydney, Sydney, New South Wales, Australia; ^6^Molecular Neurobiology Research Laboratory, Children's Medical Research Institute, The Children's Hospital at Westmead, Sydney, New South Wales, Australia; ^7^Molecular Neurobiology Research Laboratory, Kids Research and Kids Neuroscience Center, Sydney, New South Wales, Australia; ^8^Department of Biochemistry & Pharmacology, Bio21 Molecular Science and Biotechnology Institute, University of Melbourne, Melbourne, Victoria, Australia

## Abstract

Rett syndrome (RTT) is a rare, X-linked, severe neurodevelopmental disorder, predominantly associated with pathogenic variants in the methyl-CpG-binding protein-2 (*MECP2*) gene, with an increasing number of atypical RTT or RTT-like individuals having pathogenic variants in other genes, such as cyclin-dependent kinase-like 5 (*CDKL5*) or forkhead box G1 (*FOXG1*). However, ~20% of individuals with a clinical diagnosis of RTT remain genetically undiagnosed, highlighting the importance of ongoing genomic and functional studies to expand the genetic spectrum of RTT. We present a female who was born to healthy nonconsanguineous parents and presented with severe intellectual disability, macrocephaly, ataxia, absent speech, and poor eye contact. The affected individual was clinically diagnosed with atypical RTT, but genetic testing showed no pathogenic variants in *MECP2*, *CDKL5*, or *FOXG1.* Singleton whole genome sequencing was conducted, which identified a heterozygous stop–gain variant [NM_001170629.2: c.5017C>T, p.(Arg1673⁣^∗^)], in the chromodomain-helicase-DNA-binding protein 8 (*CHD8*) gene. Variant curation revealed its absence in unaffected populations, in silico predictions of pathogenicity, and an existing association with *intellectual developmental disorder with autism and macrocephaly* (*IDDAM*) (OMIM #615032). In vitro functional analyses, including Western blots, quantitative reverse transcription polymerase chain reaction (qRT-PCR), and proteomic analyses, demonstrated a significant reduction of the CHD8 transcript and two CHD8 protein isoforms in the proband's skin fibroblasts relative to control fibroblasts. Additionally, proteomic analysis indicated a significant reduction of the MeCP2 protein, indicating a possible molecular link between CHD8 and MeCP2 and thus clinically between IDDAM and RTT. As the affected individual's phenotype is consistent with atypical RTT, our results suggest that *CHD8* could be considered in the expanding genetic spectrum of atypical RTT, which may assist the diagnosis of other *MECP2*-negative RTT individuals.

## 1. Introduction

Rett syndrome (RTT) is a rare, X-linked, severe neurodevelopmental disorder, characterized by a period of developmental regression during which affected individuals lose previously acquired language and fine motor skills, including purposeful hand use. Other common hallmark features of RTT include stereotypical hand movements, acquired microcephaly, and gait abnormalities [1]. Clinically, RTT has two broad types, typical and atypical, with the latter displaying a number of clinical features seen in typical RTT while lacking key features. Typical RTT is characterized by a distinct four-stage progression pattern: normal early development (6–18 months), rapid regression, stabilization, and motor deterioration later in life (beyond age of 10) [1]. In contrast, atypical forms of RTT include less phenotypically severe cases, such as those with preserved speech (Zappella variant), the early seizure variant (Hanefeld variant), and the more severe congenital variant (Rolando variant) [1].

RTT is predominantly associated with pathogenic variants in the methyl-CpG-binding protein-2 (*MECP2*) gene, accounting for 97% of typical RTT individuals and 50%–70% of atypical individuals [1]. The MeCP2 protein mediates transcriptional repression or activation through chromatin modifications and selective binding to the methyl-CpG dinucleotide islands of target genes, which include key synaptic-related proteins involved in the glutamatergic and *γ*-aminobutyric acid (GABA)–ergic neurotransmission processes [2, 3]. Variant MeCP2 proteins disturb the finely tuned transcriptomic pattern during neurodevelopmental processes and lead to the neurological phenotypes seen in RTT [3].

“*MECP2*-negative” RTT or RTT-like individuals have been found to have pathogenic variants in cyclin-dependent kinase-like 5 (*CDKL5*) or forkhead box G1 (*FOXG1*) [4, 5]. CDKL5, located upstream of MeCP2 in the biological pathway of neuronal proliferation, maturation, and synaptogenesis, encodes a kinase that mediates MeCP2 activity via phosphorylation [6]. Conversely, *FOXG1* encodes a transcriptional repressor downstream of MeCP2 in the neurodevelopmental pathways and maintains the normal rate of neurodevelopmental processes through regulating neuronal survival/proliferation [7]. Disease-causing variants in the CDKL5 and FOXG1 proteins perturb modulation and interaction with MeCP2, leading to neurological phenotypes observed in atypical RTT [5–7]. With the accumulation of more extensive natural history data associated with pathogenic *CDKL5* and *FOXG1* variants, it was recognized that the CDKL5 deficiency disorder (CDD) and FOXG1-related encephalopathy are distinct clinical entities [7, 8].

Despite advances in sequencing technologies, up to 20% of individuals with a clinical diagnosis of RTT (i.e., ~5% of typical RTT and ~15% of atypical RTT) remain genetically undiagnosed, highlighting the importance of ongoing genomic studies to expand the genetic spectrum of RTT [1, 9]. Such studies employ several strategies, such as routinely reanalyzing existing genomic data, repeating genetic testing as new technologies emerge, and evaluating the existing RTT individuals reported with variants in genes other than *MECP2, CDKL5*, and *FOXG1*. In this context, we report an individual clinically diagnosed with atypical RTT, with a pathogenic variant in the chromodomain-helicase-DNA-binding protein 8 (*CHD8*) gene. This work outlines the identification, bioinformatic curation, and functional validation of this variant, which supports its pathogenicity and suggests the possible expansion of the genetic spectrum of atypical RTT to include *CHD8* variants.

## 2. Materials and Methods

### 2.1. Informed Consent and Biological Sample Collections

All methods used in this study were approved by the Human Research Ethics Committee of the contributing institutes with written consents obtained from the legal guardians of the proband and in accordance with the Helsinki Declaration of 1975, as revised in 2000. This family was recruited at The Children's Hospital at Westmead (Sydney, Australia). Cultured skin fibroblasts from the proband and EDTA blood from the mother were used for the studies outlined below. The father's DNA was unavailable at the time of the study.

### 2.2. Whole Genome Sequencing (WGS)

DNA was extracted from the affected individual's skin fibroblasts, and singleton WGS was performed at NATA-Accredited Victorian Clinical Genetics Services (VCGS) DNA extraction and sequencing laboratories (Melbourne, Australia).

The affected individual's WGS fastq files were analyzed by MCRI's Bioinformatics Team for quality checks, including file corruption check, quality check, and noise removal (adaptor-trimming, duplicates/contaminant sequence removal). Resultant sequences were aligned to the GRCh38/hg38 reference genome to identify regions of dissimilarity. Bioinformatic pipelines based on the Genome Analysis Toolkit (GATK) were used to conduct variant-calling. Binary Alignment Map (BAM) files storing the alignment of the affected individual's biological sequences to the GRCh38/hg38 reference genome were provided, and variant information was uploaded to the MCRI *seqr* server for *seqr*-based variant analyses [10].

### 2.3. Variant Filtering and Curation

*seqr*-based data analysis was performed on the uploaded WGS data of the affected individual [10], including variant filtering and variant curation (Table S1). Variant filtering on the *seqr* platform follows the Centre for Population Genomics (CPG) preset filters, including (1) inheritance, (2) pathogenicity, (3) annotations, (4) in silico filters, (5) frequency, (6) location, and (7) call quality, where modifications of the “Annotations” filter are detailed in Figure S1. Search 1–3 correspond to the following filter sets: (1) dominant restrictive, (2) dominant permissive, and (3) recessive restrictive. Variant curation was conducted, and the variant was classified based on the American College of Medical Genetics and Genomics (ACMG) guidelines (Table S2) [11].

### 2.4. Functional Validation

#### 2.4.1. Cell Culture

Primary fibroblast cultures were established from a skin biopsy and cultured in Dulbecco's modified Eagle medium (DMEM) with 10% fetal bovine serum (FBS), penicillin–streptomycin (P/S), and 3.9 g/L sodium bicarbonate (NaHCO_3_), at 37°C, 5% CO_2_ as previously described [12, 13]. Two control fibroblasts (C1: male, 15 years; C2: female, 2 months) were also used from healthy individuals without any suspected genetic disorders.

#### 2.4.2. Sanger Sequencing

Sanger sequencing was conducted by VCGS on DNA extracted from fibroblasts of the affected proband and the DNA extracted from blood of the clinically unaffected mother to validate the inheritance of the variant. DNA of the father was unavailable to confirm variant inheritance.

#### 2.4.3. Immunoblotting

For immunoblotting, whole cell extracts were prepared by culturing control and the affected individual's fibroblasts to confluency, rinsing twice with ice-cold PBS prior to extracting in RIPA buffer (10 mM Tris-Cl (pH 8.0), 1 mM EDTA, 0.5 mM EGTA, 1% Triton X-100, 0.1% sodium deoxycholate, 0.1% SDS, 140 mM NaCl, 1 mM PMSF and protease inhibitor cocktail (Roche)), with short gentle sonication before incubating on ice for 30 min. The cleared lysate was collected after centrifuging at 18,000*g* for 20 min at 4°C. The Pierce BCA Protein Assay Kit (Thermo Scientific #23225) was used to determine the protein concentration.

Protein lysate triplicates from each of the two control lines C1 and C2 and the affected individual line P were prepared with *β*-mercaptoethanol (bME) and protein loading dye before running sodium dodecyl sulfate–polyacrylamide gel electrophoresis (SDS-PAGE) using 4%–15% Mini-PROTEAN TGX Precast Protein Gradient Gels (BIO-RAD #4561084) in 1X running buffer at 160 V for 1 h. Proteins were transferred from SDS-PAGE gels to methanol-activated polyvinylidene fluoride (PVDF) membranes in prechilled 1X transfer buffer at 80 V for 3 h. Protein-bound PVDF membranes were blocked in 5% bovine serum albumin (BSA) blocking buffer for 1 h before primary antibody blotting overnight. A C-terminal primary antibody raised against CHD8 (Cell Signaling Technologies #11891, 1:1000) was used to quantify CHD8 protein relative to the housekeeping protein GAPDH (Sigma, #G9545, 1:5000). Subsequently, membranes were washed using 1X Tris buffered saline (TBS) with 10% Tween 20 washing buffer (1X TBST) before secondary anti-rabbit antibody (Cell Signaling #7074S, 1:5000) blotting for 1 h. Membranes were imaged with Clarity Enhanced chemiluminescence (ECL) Western Blotting Substrates (BIO-RAD#1705061) on a ChemiDoc Imaging System (BIO-RAD#12003153) and relative band intensity normalized to GAPDH using ImageLab software.

#### 2.4.4. Quantitative Reverse Transcription Polymerase Chain Reaction (qRT-PCR)

Parallel dishes were prepared with one treated with cycloheximide (CHX+) at 100 ng/*μ*L 24 h after seeding, and the other being untreated (CHX−). Forty-eight hours after seeding, cells in both CHX+ and CHX− dishes were extracted using the Isolate II RNA Mini Kit (Meridian #BIO-52072) according to the manufacturer's protocol.

RNA was extracted using the Isolate II RNA Mini Kit (Meridian #BIO-52072), following the manufacturer's protocol. Extracted RNA was used to generate cDNA using GoScript Reverse Transcriptase (Promega #A5000), following the manufacturer's protocol.

The volume of cDNA used for qRT-PCR was optimized using serial dilutions at 10, 1, 0.1, 0.01, 0.001, and 0.0001 ng/*μ*L. qRT-PCR was conducted on cDNA at 5 ng/*μ*L using PowerUp SYBR Green Master Mix for qRT-PCR (Applied Biosystems #A25742) and two sets of previously designed cDNA primers (upstream and downstream of the variant of interest), following laboratory protocols, using LightCycler 480 System (Table S3 and Figure S2).

#### 2.4.5. MS-Based Proteomic Sample Preparation Using S-Trap and Bioinformatic Analysis

In all MS-based proteomic assessments, a minimum of three independent experiments were performed simultaneously using five control fibroblast cell lines from unaffected individuals.

S-Trap sample preparation was performed according to the manufacturer's instructions (Protifi). Cell pellets were solubilized in 5% sodium dodecyl sulfate (SDS) and 50 mM tetraethylammonium bromide (TEAB, pH 8.5) and reduced and alkylated with 40 mM chloroacetamide (CAA, Sigma) and 10 mM Tris(2-carboxyethyl) phosphine hydrochloride (TCEP; Thermo Fisher Scientific) before being incubated at 99°C for 5 min with 1500 rpm shaking. Protein was digested with 1 mg of trypsin per sample (1:20 trypsin to protein ratio) at 37°C overnight. A three-step elution was performed the next day using 50 mM TEAB, then 0.2% formic acid, and lastly 50% acetonitrile (ACN). Pooled samples were dried in a CentriVap Benchtop Vacuum Concentrator (Labconco), and approximately 0.5 mg/mL of peptides was resuspended in 2% ACN, 0.1% trifluoroacetic acid (TFA) for MS analysis.

Samples were analyzed using the Label-Free Quantitation–Data Independent Acquisition (LFQ-DIA) method and a library-free approach using the Spectronaut software (REF). A database of reviewed human protein sequences containing both UniProt canonical and known isoforms was used as the search protein database (42,386 entries, downloaded 18/06/2021). BSG factory setting (default) parameters “Exclude single hit proteins,” “Major Group Top N,” and “Minor Group Top N” were unselected to allow all peptides to be considered for quantitation.

Bioinformatic analyses were completed using Perseus (v.2.0.10). Protein reports generated by Spectronaut were imported into Perseus, and potential contaminants were removed from the dataset. All raw peptide intensity counts were log_2_ transformed, and samples were grouped into either patient or control groups. All datasets were filtered to contain proteins quantified in at least two replicates for each experimental group, and then, the Student *t*-test was performed between groups with a fold change of at least 1.5 (log_2_ = 0.585) and *p* < 0.05 (>−log_10_ = 1.301) used as cut-off. Volcano plots were generated using the scatter plot option using the “Student *t*-test difference” column as *x*-axis and “−log Student *t*-test *p* value” as *y*-axis.

Enrichment analysis was conducted on proteins showing a statistically significant difference in abundance (as defined above) in proband fibroblasts compared to controls. Web-based tools, including Metascape for Gene Ontology (GO) terms and PhenoExamWeb for Human Phenotype Ontology (HPO) terms, were utilized to investigate the associated phenotypes and biological pathways [14, 15].

### 2.5. Statistical Analyses

Statistical analyses were carried out using the Mann–Whitney test for CHD8 isoform quantity, Wilcoxon test for CHD8 transcript quantity (GraphPad Prism Software, v9.1.1, San Diego, California, United States), and Student's *t*-test for MS-based proteomic assessments; error bars represent the standard error of the mean (±SEM) and *p* < 0.05 (and for proteomic data fold change > 1.5) was considered as statistically significant. For all quantitative analyses, a minimum of three independent experiments were performed.

The Mann–Whitney test was used as the CHD8 isoform abundance is continuous and not normally distributed across the independent proband and control groups. The Wilcoxon test was used to quantify the relative change of CHD8 transcripts in the proband (CHX+/− paired group) and the controls (CHX+/− paired group).

## 3. Results

### 3.1. Case Description

A female individual (now 23 years of age) born following an uneventful pregnancy to healthy, nonconsanguineous parents presented with language regression, global developmental delay, severe intellectual disability, macrocephaly, poor sleep initiation, bruxism, poor eye contact, inappropriate screaming spells, generalized tonic–clonic seizures, intermittent hyperventilation, hypotonia, ataxia with broad-based gait, and stereotypic hand flapping/clapping movements. With the exception of macrocephaly, the clinical phenotype of the affected individual was consistent with a clinical diagnosis of atypical RTT as per the revised Neul's diagnostic criteria for RTT (Table S4) [1].

### 3.2. Variant Details and Curation

As part of the genetic diagnosis for the patient with RTT-like features, sequencing of the *MECP2*, *CDKL5*, and *FOXG1* genes, including multiplex ligation-dependent probe amplification studies of *MECP2*, at the Molecular Genetics Department, Children's Hospital at Westmead, did not identify any sequence variants, deletions or duplications. Our analysis of singleton WGS data identified a heterozygous, stop–gain variant: *[GRCh38(chr14): g.21866016G>A; NM_001170629.1: c.5017C>T; NP_001164100.1: p.(Arg1673*∗*)]* in the (*CHD8*) gene.

**Figure 1:**
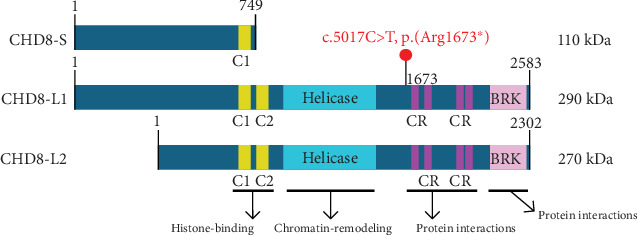
Isoforms and functional domains of the chromodomain-helicase-DNA-binding protein 8 (CHD8) protein. The three isoforms of CHD8 protein, including (1) CHD8-S, a short isoform; (2) CHD8-L1, a long isoform; and (3) CHD8-L2, a long isoform [17]. CHD8-L1 and CHD8-L2 are composed of two histone-binding chromodomains (C1 and C2, yellow), a chromatin-remodeling helicase domain (helicase, cyan), multiple protein-interacting chromatin organization modifier domains (CR, magenta), and a DNA-binding brahma and kismet domain (BRK, pink) [17]. The position of the identified variant relative to CHD8-L1 and CHD8-L2 isoforms is indicated in red.

The CHD8 protein is an ATP-dependent chromatin remodeling factor and has shown function in processes including transcriptional regulation, epigenetic remodeling, promotion of cell proliferation, and regulation of RNA synthesis [16]. Alternative splicing of CHD8 results in three predominant, ubiquitously expressed protein isoforms, including (1) CHD8-S, a short isoform with a molecular weight of ~110 kDa; (2) CHD8-L1, a long isoform with a molecular weight of ~290 kDa; and (3) CHD8-L2, a long isoform with a molecular weight of ~270 kDa (Figure 1) [17]. The two long isoforms are composed of two histone-binding chromodomains, a chromatin-remodeling helicase domain, multiple protein-interacting chromatin organization modifier domains, and a DNA-binding brahma and kismet (BRK) domain [17].

The identified variant substitutes a thymine for a cytosine at the 5017th base position (c.5017T>C), converting an arginine to a stop codon at the 1673rd amino acid position (p.Arg1673∗) of isoform CHD8-L1 (Figure 1). This variant is predicted to induce nonsense-mediated decay (NMD) of the CHD8-L1 and CHD8-L2 transcripts, and loss of function variants in *CHD8* are associated with *intellectual developmental disorder with autism and macrocephaly* (IDDAM) (OMIM #615032) The variant is absent in unaffected populations listed in gnomADv2-4, with one ClinVar entry classifying it as *likely pathogenic* (LP) (ClinVar ID: 2439084) and one LOVD entry classifying it as *pathogenic* (CHD8_000032). It shows high conservation across all vertebrates, and the in silico tool (i.e., pLi = 1) predicted it to be intolerant to loss of function type of variants. Additionally, 100 pathogenic NMD variants have been reported in the *CHD8* gene by ClinVar. Therefore, based on the ACMG criteria [11], this variant is classified as *pathogenic* (PVS1_with partial phenotype match, PM2_supporting, PP3). Given the clinical diagnosis of atypical RTT, we undertook functional studies to support the pathogenicity of the variant.

### 3.3. Sanger Sequencing and qRT-PCR

**Figure 2:**
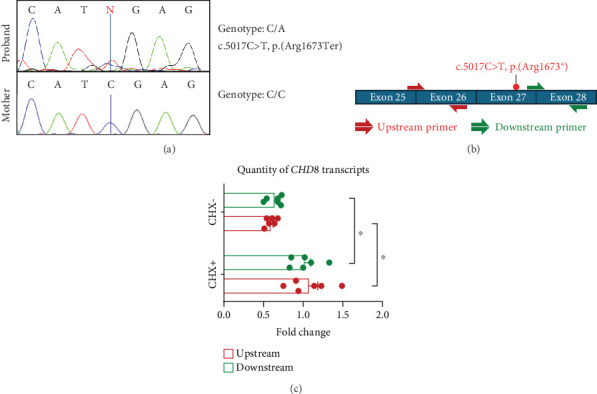
Variant validation using Sanger sequencing and quantitative reverse transcription polymerase chain reaction (qRT-PCR). (a) The Sanger chromatograms indicate the absence of the variant in the maternal DNA and presence in the proband fibroblasts and blood DNA, indicating a nonmaternal inheritance of the variant. (b) Two sets of cDNA primers, including a set of primers upstream of the variant and another downstream of the variant (Table S3 and Figure S2), were used to conduct qRT-PCR on *CHD8* cDNA in the proband line versus the control lines. (c) *CHD8* transcripts captured by both upstream and downstream cDNA primers showed significant reduction (upstream primers: ~42%, downstream primers: ~33%) in the CHX− proband samples relative to that of controls (Wilcoxon test: *p* = 0.0313 for both primer sets). CHX+ samples of both the proband and the controls showed equivalent levels of *CHD8* transcripts.

Sanger sequencing of fibroblast DNA from the proband confirmed the variant, which was not present in blood DNA from the mother (Figure 2a). The father's DNA was unavailable to confirm the segregation. qRT-PCR analysis on cDNA generated from CHX+/CHX− samples of the proband's and control fibroblasts confirmed that the CHX− proband samples exhibited approximately half of the *CHD8* transcript levels compared to the CHX− control samples (upstream primer: 42%, *p* = 0.0313; downstream primer: 33%, *p* = 0.0313), whereas the CHX+ samples of both the proband and the controls showed equivalent levels of *CHD8* transcripts suggesting the transcript is undergoing NMD (Figure 2b,c).

### 3.4. Immunoblotting and Mass Spectrometry–Based Proteomic Analysis

**Figure 3:**
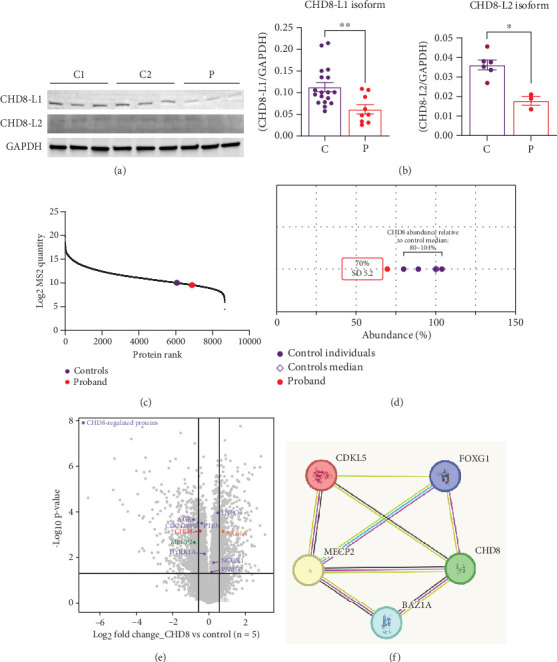
Immunoblotting and mass spectrometry–based proteomic analysis. (a) Western blots indicating the level of CHD8 protein detected from controls (C1, C2) and proband (P) samples. Three technical repeats (*n* = 3) of Western blotting using the CHD8 C-terminal antibody (Cell Signaling Technologies #11891, 1:1000) showed the relative quantities of CHD8-L1 and CHD8-L2 against GAPDH (loading control). (b) Protein band quantification of the Western blots showed a significant reduction of the CHD8-L1 and CHD8-L2 isoform levels in the proband (P) (L1: ~51%, L2: ~48%) compared to those of the controls (C) (Mann–Whitney test: *p* = 0.0089, *p* = 0.0238, respectively). (c) The abundance of CHD8 is ranked significantly lower in the proteome of the proband compared to the controls. (d) The abundance of CHD8 is significantly lower in proband fibroblasts (70%, red dot) and lies outside of the control range (80%–104%, *n* = 5). (e) Volcano plot showed the relative amount of proteins in the proband line compared to the controls, with vertical lines indicating +/−1.5 log_2_-fold change and the horizontal line indicating statistical significance. CHD8 is reduced significantly by ~30% (*p* < 0.001) in the proband line compared to the controls. MeCP2 (green) is significantly reduced by ~43% (*p* < 0.01), whereas bromodomain adjacent to zinc finger domain 1A (*BAZ1A*) encoding the accessory subunit of the ATP-dependent chromatin assembly factor (ACF) (orange) is significantly increased by ~72% (*p* < 0.001). CHD8-regulated proteins (purple), including acylglycerol kinase (AGK), CDC42-binding protein kinase (CDC42BPB), phosphatase and tensin homolog (PTEN), and dual-specificity tyrosine phosphorylation-regulated kinase 1A (DYRK1A), showed a reduction in their corresponding protein abundance, with AGK being the highest at ~55% (*p* < 0.001). Transportin 3 (TNPO3), nuclear receptor corepressor 1 (NCOR1), and proteasome assembly chaperone 2 (PSMG2) showed an increase of abundance with TNPO3 being the highest at ~39% (*p* < 0.001). (f) STRING network analysis revealed coexpression (black), interactions (magenta), and comentions in literature (lime green) between CHD8, MeCP2, CDKL5, FOXG1, and ACF.

Western blotting using the C-terminal antibody confirmed a significant reduction of the CHD8-L1 (~51%, *p* = 0.0089) and CHD8-L2 (~48%, *p* = 0.0238) isoforms in the proband skin fibroblasts compared to the controls (Figure 3a,b). All detectable proteins in the fibroblasts of both the proband and the controls were ranked based on their log_2_ MS2 quantity (Figure 3c). A higher log_2_ MS2 value corresponds to a more highly expressed protein. CHD8 was shown as a highly abundant protein in control fibroblasts and was found at lower abundance in the proband's fibroblasts. Next, we calculated the median level of protein expression in controls and defined the normal range of CHD8 protein abundance as 80%–104% (Figure 3d). MS-based proteomic analysis demonstrated a significant (~30%) reduction of the CHD8 protein (identified by shared peptides arising from both CHD8-L1 and CHD8-L2 isoforms) in the proband skin fibroblasts compared to the controls (*p* < 0.001) (Figure 3e) (Table S5). Among detectable CHD8-regulated proteins [18], acylglycerol kinase (AGK), CDC42-binding protein kinase (CDC42BPB), phosphatase and tensin homolog (PTEN), and dual-specificity tyrosine phosphorylation-regulated kinase 1A (DYRK1A) all demonstrated reduction in their protein abundance, with AGK showing the most significant reduction by approximately 55% (*p* < 0.001) compared to controls. In contrast, transportin 3 (TNPO3), nuclear receptor corepressor 1 (NCOR1), and proteasome assembly chaperone 2 (PSMG2) demonstrated an increase in abundance, with TNPO3 showing the largest increase at around 39% (*p* < 0.001). Protein complex partners and binding partners of CHD8 were also investigated and there was no significant difference in the abundance of these proteins in the proband fibroblasts compared to controls (Table S5). Interestingly, MeCP2 was significantly reduced by ~43% (*p* < 0.01). STRING analysis (medium confidence: 0.400, high stringency: 1%) indicated that CHD8 is directly related to MeCP2, CDKL5, and FOXG1 (Figure 3f). CHD8 is also indirectly associated with it through the bromodomain adjacent to zinc finger domain 1A (*BAZ1A*) gene. Interestingly, CHD8, MeCP2, and the *BAZ1A*-encoded ATP-dependent chromatin assembly factor (ACF) showed coexpression and molecular interactions with each other. However, unlike the significantly downregulated MeCP2, ACF showed a significant increase by ~72% (*p* < 0.001) (Figure 3e). The abundance of CHD8 protein in the proband fibroblasts was approximately 70% compared to the control levels, falling outside the normal range, consistent with the 30% reduction being degradation of the protein encoded on the mutated allele, as predicted by NMD for this nonsense variant.

**Figure 4:**
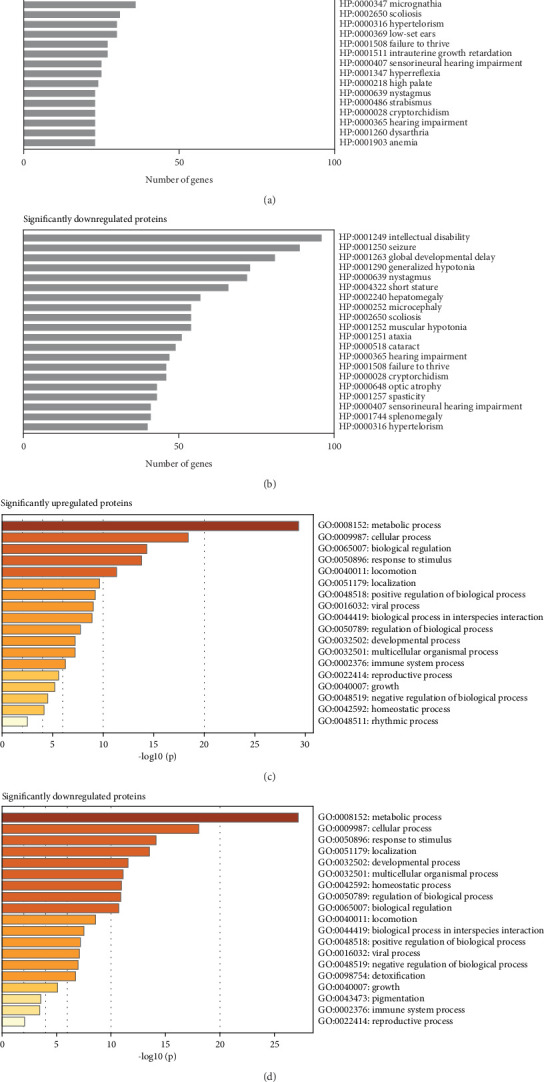
Enrichment analysis on mass spectrometry–based proteomic analysis data. Enrichment analysis was conducted on proteins with a statistically significant change of abundance in the proband fibroblasts compared to the controls (Table S5). PhenoExamWeb was utilized to identify associated Human Phenotype Ontology (HPO) terms of the significantly (a) increased and (b) reduced proteins [14], and Metascape was utilized to investigate the associated Gene Ontology (GO) terms of the significantly (c) increased and (d) reduced proteins [15].

Subsequent gene set enrichment analysis of the 625 significantly increased proteins and 751 significantly reduced proteins in the proband samples relative to controls was undertaken (Table S5). Several enriched HPO terms are consistent with the proband's phenotype, such as global developmental delay, seizures, intellectual disability, ataxia, and hypotonia (Figure 4a,b). In comparison, the enriched GO terms lack disease specificity and highlight general biological processes rather than unique pathways linked to the proband's phenotypes (Figure 4c,d).

## 4. Discussion

We identified *CHD8* as a novel candidate gene associated with atypical RTT by validating a pathogenic variant in this gene at the genomic, transcript, and proteomic level. Evidence from Sanger sequencing confirmed the presence of this pathogenic variant in *CHD8 [NM_001170629.2: c.5017C>T, p.(Arg1673*∗*)]*, while qRT-PCR analysis revealed a significant reduction of CHD8 transcripts due to NMD. This was further supported by immunoblotting evidence and MS-based proteomic analysis, which also identified a significant change in the RTT-associated MeCP2 protein and its common protein partner, ACF [19]. This suggests the possible link between *CHD8* variants and the atypical RTT phenotype in the individual reported here, which represents a potential expansion of the phenotypic spectrum of *CHD8* and the genotypic spectrum of atypical RTT.

Previous CHD8 research has primarily focused on its involvement in IDDAM, which is characterized by intellectual disability, developmental regression, seizures, sleep disturbances, autism, and overgrowth [20]. The condition was first reported in 2011 among a group of individuals with sporadic autism spectrum disorder (ASD) [21], and the characterization of CHD8 functions facilitated the establishment of an association between *CHD8* variants and IDDAM. CHD8 is an ATP-dependent chromatin remodeling factor that regulates the rate of transcription through interacting with epigenetic markers, modulating chromatin accessibility, and adjusting the expression of long noncoding RNAs (lncRNAs) [22–25]. CHD8 binds to euchromatin regions with epigenetic H3K4me3 marks (i.e., transcriptionally permissive) and increases the accessibility of these genes, allowing the binding of RNA polymerase II and other transcription factors to initiate transcriptions [23, 26, 27]. On the other hand, CHD8 also interacts with epigenetic factors to regulate H3K36 methylation levels and influence alternate splicing [24]. Finally, CHD8 has been shown to both positively and negatively regulate the expression of specific lncRNA genes, further indicating that CHD8 has diverse roles in transcriptional regulation [25]. Interestingly, the intricate orchestration of gene expression by CHD8 has been shown to follow a nonlinear, dosage-dependent pattern, indicating that there remain other mechanisms, such as competitive transcription factor binding, involved in transcriptional regulations [23, 28].

Expression studies showed that CHD8 knockdown disrupts the finely tuned expression pattern of genes involved in neurodevelopmental processes, such as axon guidance, neurotransmitter secretion, dendrite morphogenesis, and synaptic development [18, 28, 29]. Additionally, CHD8 depletion has also shown consequences on more general cellular processes, such as DNA damage repair, DNA replication, and cell adhesion, which are possibly involved in regulating the proliferation of cortical progenitor cells external to the ventricular zone of the human brain [22, 28, 30]. In rodent models, *Chd8* haploinsufficiency causes a global dysregulation of gene expression, leading to impaired neurogenesis, neuronal connectivity, and neuronal differentiation [22, 29, 31]. The neurological implications are also reflected as behavioral phenotypes, including but not limited to repetitive stereotypies, increased anxiogenic and depressive symptoms, and reduced social interactions [31, 32]. These are consistent with the neurological and behavioral symptoms seen in IDDAM but notably are also often seen in RTT^1^. In fact, the gait abnormalities, stereotypic hand movements, breathing disturbances, bruxism, and inappropriate screaming spells seen in the affected individual reported here are more commonly reported in RTT than in IDDAM^1^. This suggests that the *CHD8* variant described in this study may lead to an atypical RTT clinical presentation. We do note, however, that the macrocephaly in the individual reported here is more in keeping with IDDAM. Acquired microcephaly is more commonly seen in RTT^1^, although macrocephaly has also been reported [33]. Additionally, proteomic studies on the skin fibroblasts of the proband also observed a widespread downregulation of proteins, supporting the possibility of a specific pattern of dysregulation of gene expressions seen in *CHD8* haploinsufficiency [32]. Therefore, the current findings align with the existing evidence while offering a novel perspective on the association of *CHD8* with atypical RTT.

Considering such a pivotal role of CHD8 in neurodevelopment, we propose that *CHD8* variants may contribute to the pathogenesis of atypical RTT in the individual reported here by dysregulating the transcription pattern of key brain-developing genes, possibly via influencing the expression of the RTT-associated protein, MeCP2 [1]. Through STRING analysis, CHD8 showed comentions in the literature, coexpression, and experimentally determined interactions with MeCP2 (Figure 3f) [19]. As a well-established transcriptional modulator [2], MeCP2 does so through interacting with epigenetic markers [34], noncoding RNAs [35], and corepressor proteins to induce chromatin modifications and DNA methylation [36], which resemble the molecular functions of CHD8. Additionally, direct interactions between homologs of MeCP2 and CHD8 have been described in mice and fruit flies [19, 37–40]. This is further supported by our observation of the significant downregulation of MeCP2 when CHD8 is reduced and a previous study that showed *CHD8* as one of the most differentially expressed genes in a mouse *Mecp2* knockdown model [41]. CHD8 could also interact with MeCP2 through its association with an interacting partner, ACF, which is an accessory subunit of ATP-dependent chromatin remodeling factor that plays a crucial role in regulating gene expression [42]. Previous reports have indicated that ACF interacts with CHD8 in yeast homologs (*Saccharomyces cerevisiae*) [43, 44] and with MeCP2 in humans [45, 46], suggesting a potential link between CHD8 and MeCP2 through ACF. Notably, CHD8 also shows coexpression with CDKL5 and interactions with FOXG1 (*Saccharomyces cerevisiae*) [47, 48], which could indicate an additional pathway of pathogenesis in producing RTT-like phenotypes.

There are limitations to the current study. The enrichment analysis on differentially expressed proteins should be interpreted with caution, as, in our experience, the enriched HPO terms include common phenotypes frequently associated with a wide range of genetic disorders. The broad functional roles and ubiquitous expression of CHD8 may explain the nondisease-specific GO terms enriched in this dataset. Therefore, these limitations inherent in analyzing a single case made it challenging to draw meaningful conclusions from the enrichment analysis. Future studies should focus on expanding the cohort to include additional RTT-like cases with *CHD8* variants to identify disease-specific GO terms and improve the significance of the enrichment analysis.

The currently described variant has been previously reported with phenotypes dissimilar from the current affected individual, including the lack of gait abnormalities, stereotypies, and social behaviors [49]. Given the nonlinear, dosage-sensitive relationship of gene expression regulation by CHD8 [28], it is possible that there are other genetic/nongenetic factors in the previously reported individuals and the subject of this report that influence the CHD8 level (e.g., possible differing levels of NMD of CHD8 transcripts), potentially leading to varying consequences of gene dysregulation. This could also explain the association of the current variant with RTT-like clinical features, whereas most other *CHD8* variants are associated with the IDDAM phenotype. Ultimately, this presumption requires further investigation in individuals of different genetic background(s) with the different *CHD8* variants to explore the relationship between the expression level of CHD8 and its downstream effects on gene expression and disease phenotype. The current study only investigated the in vitro consequences of the currently described variant with a specific focus on the CHD8 protein itself. Future investigations could include studies to explore the biological pathways involved and establish potential functional associations with MeCP2, ACF, and atypical RTT symptoms.

## 5. Conclusion

This study reports a *MECP2-*negative atypical RTT individual with a pathogenic variant in the *CHD8* gene. Functional validation of the variant confirmed a significant reduction of CHD8 protein due to NMD of the variant *CHD8* transcripts. Current evidence linking CHD8 with RTT-associated genes is limited and primarily based on in silico analysis, and more robust functional validation is necessary to establish a potential association between *CHD8* and atypical RTT. With further supporting evidence, *CHD8* could be considered as a candidate for screening in *MECP2*-negative atypical RTT individuals.

## Data Availability

The data that support the findings of this study are available from the corresponding author upon reasonable request.

## General Statement

*Citation Standards*. All data, program code, and other methods have been appropriately cited. We recognize them as original intellectual contributions and afforded recognition through citation.

## Supporting Information

Supporting Information Additional supporting information can be found online in the Supporting Information section. The following supporting information is included: Detailed case report. Detailed next-generation sequencing methods. Table S1. Whole genome sequencing data analysis pipeline with *seqr*-based curation and complementary manual curation. Table S2. Manual curation sites and the respective information obtained. Table S3. *CHD8* cDNA primer sequences. Table S4. Clinical profile of the atypical RTT case per Neul's revised clinical diagnostic criteria for RTT^1^. Table S5. Mass spectrometry–based proteomic analysis and enrichment analyses of Human Phenotype Ontology and Gene Ontology terms. Figure S1. Variant annotation search levels. Figure S2. Location of cDNA primers designed for *CHD8* qRT-PCR.
