# Supplementary material for: CHD8 Variant and Rett Syndrome: Overlapping Phenotypes, Molecular Convergence, and Expanding the Genetic Spectrum
Source: Hum Mutat. 2025 Jun 2;2025:5485987. doi: 10.1155/humu/5485987 (PMC12149508; doi:10.1155/humu/5485987)
Supplement: Supporting Information — Additional supporting information can be found online in the Supporting Information section. The following supporting information is included: Detailed case report. Detailed next-generation sequencing methods. Table S1. Whole genome sequencing data analysis pipeline with seqr-based curation and complementary manual curation. Table S2. Manual curation sites and the respective information obtained. Table S3. CHD8 cDNA primer sequences. Table S4. Clinical profile of the atypical RTT case per Neul's revised clinical diagnostic criteria for RTT1. Table S5. Mass spectrometry–based proteomic analysis and enrichment analyses of Human Phenotype Ontology and Gene Ontology terms. Figure S1. Variant annotation search levels. Figure S2. Location of cDNA primers designed for CHD8 qRT-PCR. [file 5485987.f1.zip › ElaineZ_CHD8_paper_Supplementary Material_Figures_TablesS1-4_funding&COIadded.docx]

## *Supplementary Materials*

### Detailed case report

The proband was born at 38 weeks gestation by normal delivery. She required oxygen for a short period after birth (10-15 minutes) and was observed in Special Care Nursery overnight. She had bilateral cephalohaematoma and had jaundice requiring phototherapy for 2-3 days. Her birth weight was 3.5kg (74th percentile), length 53cm (98th percentile), and head circumference 34 cm (51st percentile). Her parents first became concerned about her development at approximately six months of age when she was not able to roll. She sat unsupported at 14 months, pull to stand at 17 months and learnt to walk at 19 months. As an infant she was not interested in playing with toys and did not reach out or hold an object until approximately 12 months, but did enjoy music. She developed pincer grip after her first birthday and learnt to drink from a cup after two years of age. In addition to her delayed development, there were also concerns of developmental regression. She babbled at 4-5 months but when reviewed at 46 months she vocalised and was not babbling. She also learnt to say “wow” but stop doing that after 2 years. She used to pat her abdomen to indicate she was hungry at one year of age but stopped doing so at two years. After two years of age, she developed stereotypic hand movements in the form of hand flapping, clapping, tapping her face and her mouth with her hands, and hitting her head. Her development was assessed using Griffith Mental Developmental Scale at 45.3 months of age which showed severe to profound global developmental delay. She was able to finger-feed but often after one bite drops food to the floor. She drank from a cup which she can put down neatly on a table but at other times drop it on the floor. She was not toilet trained and could not indicate if the nappy needs changing. She communicated by pulling her parents by the hand, to for example, the fridge when hungry. Her vocalisation was limited consist of repetitive words such as “ticker, ticker”. Words such as “mama” and “papa” were not used in context. She did not respond to her name when being called, although audiology assessment was normal. She had no difficulties chewing and swallowing most food, but meat needs to be cut into small pieces. She walked independently with a broad-based gait. She walked upstairs holding an adult’s hand and placing both feet on the same step, but unable to navigate stairs independently. She was not interested in rolling, throwing or catching a ball. Her finger movements were very agile and that she was able to pick up small items. Her play skills were limited, although she enjoyed her music box. She enjoyed ripping pieces of paper and flicking her fingers in front of her eyes. She had bruxism, occasional constipation, and a disturbed sleep pattern with nocturnal crying.

The Griffith Mental Developmental Assessment was repeated at 5 years and 10 months of age. It was noted at this stage that she had some interval developmental gains. She walked short distances, able to climb stairs holding the rail, and sometimes run in the playground. She was able to feed herself when hungry and can take the top of yoghurt containers. She fed herself predominantly using fingers but was able to use spoon. She was also able to make choices between foods that she liked. She mouthed a lot of objects such as shoes, leaves, and dirt in her mouth. Her communication was limited and had not developed spoken words. She moved towards an adult when distressed but was not able to share happiness. She cried when angry or frustrated. She was able told a pen and may make some a scribble. This assessment result was consistent with previous findings of severe/profound global developmental disability.

There was a concern about possible seizures after 5 years of age when she developed episodes where she shook her head and held her hands rigidly to the side. There were also reports of staring episodes. Electroencephalogram (EEG) performed did not show definite focal features or epileptiform discharges. She subsequently had two episodes of generalised tonic-clonic seizures. An EEG performed at that time did not show epileptiform activity, however, there was diffused cerebral dysfunction. She was started on sodium valproate graded up to 400 mg BD. She was otherwise in good health and occasionally has constipation which is managed with increased fluid intake and fibre.

She was last reviewed at age 19 years. She was described as a fussy eater and has lost some weight. She had been diagnosed with *Helicobacter pylori* enteritis but did not have any difficulty chewing, swallowing, or any history to suggest aspiration. She had good hand function, was able to finger feed, use a fork to feed herself noodles, and use a spoon for small pieces of food. She was also able to use an iPad. Her constipation had been well managed with increased fluid, fibre and physical activities. She remained active with independent mobility and was able to walk all day without fatigue or assistance. She had also reached her adult height when reviewed. Her height was 181.9cm (99th centile, Z score of 2.89), her weight was 58.1kg (53rd centile, Z score of 0.08), and the head circumference was 57cm (99^th^ percentile). She was alert and cooperative throughout the assessment. She did not vocalise. Cardiovascular, respiratory and abdominal examinations were normal. She had normal muscle tone and no joint contractures. She was able to walk unassisted with a steady gait. She held a toy with both hands in the midline and constantly played and twisted it. She was able to open a zippered bag and remove objects from the bag. She had truncal rocking in the seated position and tended to hunch forward. Blood collected after her assessment showed low ferritin level. She was commenced on iron supplementation. ECG did not show cardiac conduction abnormalities.

Her genetic investigations included negative *MECP2* screening (exon 1-4) and normal MLPA testing Next generation sequencing (NGS) of an epileptic encephalopathy gene panel (TruSight One panel) identified a heterozygous missense variant of uncertain significance in Chromodomain-helicase-DNA-binding protein 2 [*CHD2*; chr15:93558040C>A; NM_001271.3:C.4807C>A; p.(Pro1603Thr)]. Various *in silico* tools consistently predicted this variant to be benign [Mutationtaster: polymorphism (p = 0.693), PolyPhen-2: benign (p=0.025), SIFT: tolerated (score = 0.756; cutoff = 0.05), Grantham score = 38]. Other metabolic investigations were normal including serum transferrin isoforms, plasma very long chain fatty acids, plasma homocysteine, urine amino and organic acids, urine purines and pyrimidines, urine glycosaminoglycans, and white blood cell lysosomal enzymes.

### Detailed Next Generation Sequencing methods

WGS libraries were prepared using Nextera™ DNA Flex Library Prep and loaded onto a HiSeq 4000 sequencer (Illumina; HiSeq control Software v3.4.0) and 2 x 150 bp paired-end sequencing was performed at the Translational Genomics Unit, Victorian Clinical Genetic Services (VCGS). Samples passed sequencing QC with > =80% bases with at least Q30, and mean coverage of at least 27-fold (aim 30-fold). Raw sequencing data was converted to fastq format using Illumina's bcl2fastq2 converter (v2.17.1.14). Data was processed using Cpipe (http://cpipeline.org/; version 2.3.0)^50^, in order to generate annotated variant calls within the target region (coding exons +/- 2bp), via alignment to the reference genome (GRCh37; hg19) and a total of 25265 variants were found. Candidate variants identified through this analysis were not found to be associated with the proband’s phenotype through functional and clinical validations (data available on request).

### Funding statement for production

This research was supported by an Australian National Health and Medical Research Council (NHMRC) Investigator Fellowship (GNT2009732 to D.A.S.) and the Australian Medical Research Future Fund (MRFF) Genomics Health Futures Mission (MRF2016030 to D.A.S. and J.C.). We thank the Mito Foundation for the provision of instrumentation through research equipment grants to D.A.S. The research conducted at the Murdoch Children's Research Institute (MCRI) was supported by the Victorian Government's Operational Infrastructure Support Program. The Chair in Genomic Medicine awarded to J.C. is generously supported by The Royal Children's Hospital Foundation.

### Conflict of interest statement for production

The authors declare no conflicts of interest in preparing this article.

### Supplementary figure/tables

Table S1. Whole genome sequencing data analysis pipeline with *seqr-*based curation and complementary manual curation.

| *Seqr* variant search filters | | | | | | |
| --- | --- | --- | --- | --- | --- | --- |
| *Filters* | *Search 1* | | *Search 2* | *Search 3* | P: Pathogenic  LP: Likely pathogenic  VUS: Variant of Uncertain Significance  w/o: Without  UTR: Untranslated Regions  AF: Allele frequency  GQ: Genotype quality  AB: Allele balance  WES: Whole exome sequencing  WGS: Whole genome sequencing SVQ: Structural variant quality |  |
| Inheritance | *De novo* / dominant | | As 1 | Recessive |  |  |
| Pathogenicity | ClinVar: P, LP | | As 1 + VUS | As 1 |  |  |
| Annotations | Level 1 | | Level 2 | Level 1, w/o UTR, promoter |  |  |
| *In silico* filters | None | | None | None |  |  |
| Frequency | AF: 0.001, Callset AF: 0.01 | | AF: 0.001, Callset AF: 0.1 | AF: 0.01, Callset AF: 0.03 |  |  |
| Location | None | | None | None |  |  |
| Call quality | GQ: 20, AB: 20, WES SVQ: 50, WGS SVQ: 10 | | GQ: 20, AB:10, WES SVQ: 20 | As 1 |  |  |
| Complementary manual curation | | | | | | |
| 1. *Variant annotation* | | *4. Protein analyses* | | | *6. Literature search* |  |
| - Available on *seqr* | | - Mutational spectrum - Gene-level and regional constraint (site of mutation) - Protein domains and functions - Key functional domains, binding sites, post-translational modifications, motifs, structures, and topography - Cellular localisation | | | - Previously reported cases, and associated variants identified - Transcriptional studies, 3D modelling studies, functional studies, and segregation analyses - Explore the molecular biology of gene-of-interest - Establish disease pathogenesis and link with identified variants |  |
| *2. Gene curation* | |  |  |  |  |  |
| - Gene-disease association - Disease mechanisms - Mutational spectrum - Function and expression | |  |  |  |  |  |
| *3. Population frequency* | | *5. Variant annotation* | | |  |  |
| - Exome/genome sequencing coverage - Alternative residue | | - Amino acid conservation via UCSC (University of California, Santa Cruz) browser - Other in silico predictions unavailable in seqr (e.g., Grantham score, AlphaMissense) | | |  |  |

Notes: Variant curation on *seqr* followed the Centre of Population Genomics (CPG) pre-set filters including: (1) inheritance; (2) pathogenicity; (3) annotations; (4) in silico filters; (5) frequency; (6) location; and (7) call quality, where the modification of the ‘Annotations’ filter is detailed in **Supplementary materials: Figure S1**. Search 1 – 3 correspond to the following filter sets: (1) dominant restrictive; (2) dominant permissive; and (3) recessive restrictive. Variant curation was conducted, and the variant was classified based on The American College of Medical Genetics and Genomics (ACMG) guidelines (**Supplementary materials: Table S2**).


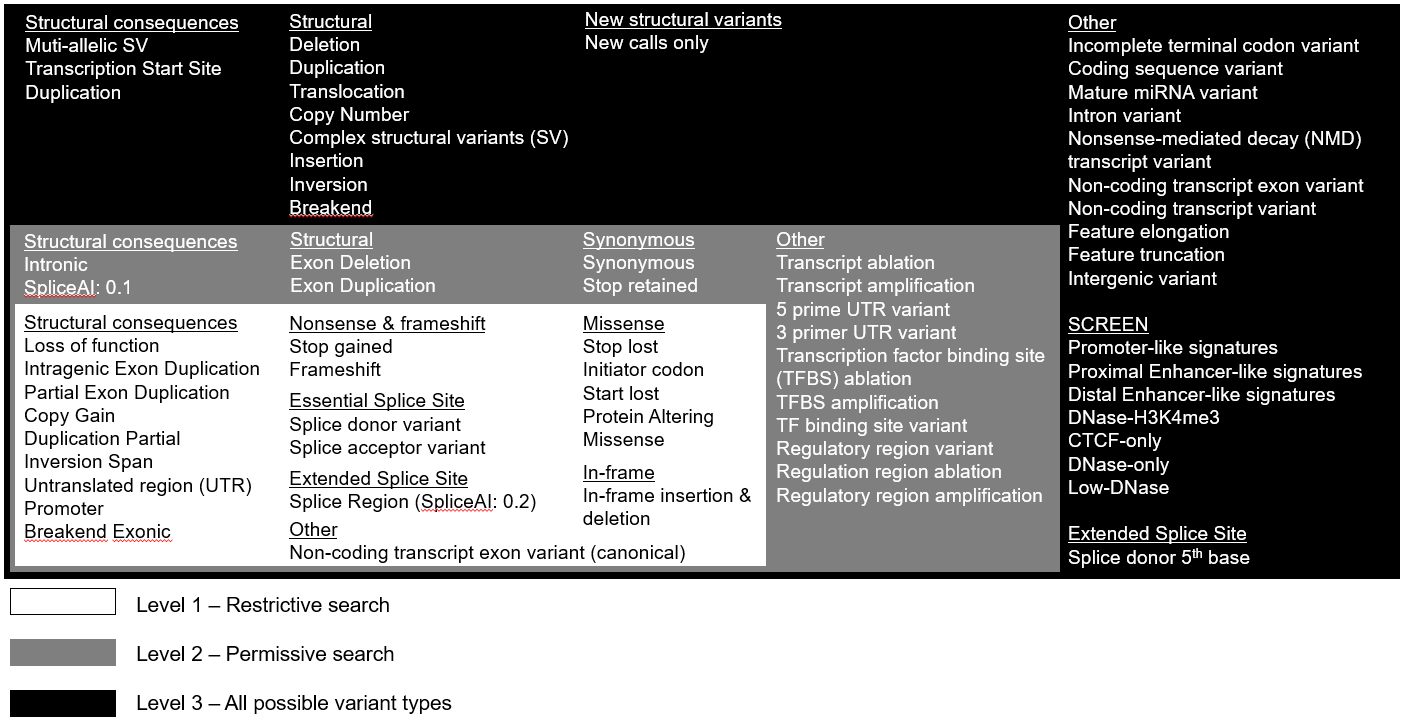


**Figure S1. Variant annotation search levels.** Pre-set filters designed by CPG, outline the consequences of variants in 3 levels. Level 1 depicts the consequences that severely affects protein structure/function, and thus are the most likely to be pathogenic. Level 2 depicts consequences with relatively easy interpretation, and level 3 depicts those with challenging interpretations.

Table S2: Manual curation sites and the respective information obtained.

| Manual curation | Information obtained |
| --- | --- |
| [NCBI Gene](https://www.ncbi.nlm.nih.gov/gene/) | - Gene name, direction, genomic location (hg38/hg19); transcripts |
| [ClinVar](https://www.ncbi.nlm.nih.gov/clinvar/) | - Disease-relevant transcripts - Gene-disease association, mutational spectrum |
| [Decipher](https://www.deciphergenomics.org/) | - Relative position of variants from functional domains - Regional constraint at the site of mutation |
| [PubMed](https://pubmed.ncbi.nlm.nih.gov/) | - Transcriptional studies, functional studies, and segregation analyses - Molecular biology of the gene-of-interest |
| [GTEx portal](https://gtexportal.org/home/) | - Expressions (tissue-specific, exon-specific, etc) of every gene transcript |
| [UniProt](https://www.uniprot.org/) | - Protein functions, domains, binding sites, post-translational modifications, key structures, topography, and cellular localisation |
| [InterPro](https://www.ebi.ac.uk/interpro/) | - Functions of key protein domains/families/structures |
| [gnomAD](https://gnomad.broadinstitute.org/) | - Number of unaffected individuals with the variant-of-interest - Alternative/nearby nucleotide/residue of the variant-of-interest - Whole gene constraints (missense, loss of function constraints) |
| [UCSC browser](https://genome.ucsc.edu/) | - Nucleotide/amino acid conservation across 100 species |
| [OMIM](https://www.omim.org/) | - Gene-disease associations, gene functions - Past cases/variants reported, phenotype/variant spectrum - Disease inheritance, penetrance/expressivity, severity, onset |
| [KEGG](https://www.genome.jp/kegg/) | - Molecular and biological pathways associated with the gene of interest |
| [MGI](https://www.informatics.jax.org/) | - Mutant mouse information (genetic, genomic, and biological data) |
| [CADD](https://cadd.gs.washington.edu/) | - Grantham score |
| [HOPE](https://www3.cmbi.umcn.nl/hope/about/) | - Protein structure modelling, prediction of missense consequences |
| [DynaMut](https://biosig.lab.uq.edu.au/dynamut/) | - Prediction of the impact of mutation(s) on protein stability, dynamics, flexibility |
| [Missense3D](http://missense3d.bc.ic.ac.uk/) | - Protein structure modelling, prediction of missense consequences |
| [AlphaMissense](https://alphamissense.hegelab.org/) | - Prediction of missense consequence |
| [AlphaFold](https://alphafold.ebi.ac.uk/) | - Protein structure visualisation |
| [MetaDome](https://stuart.radboudumc.nl/metadome/) | - Mutation tolerance |
| [STRING](https://string-db.org/) | - Protein-protein interaction networks, functional enrichment analysis |
| [Mastermind](https://mastermind.genomenon.com/) | - Empirical evidence - Genetic variant Interpretation |

| Primer | Sequence (5’ to 3’) | Reverse compliment | Product length | GC% | Tm | Self-complementarity | Self-3’complementarity |
| --- | --- | --- | --- | --- | --- | --- | --- |
| cDNA_1F | GCGATTGCCAGTGAGATTGAC |  | 105 | 52.38 | 59.94 | 4 | 2 |
| cDNA_1R | GACTCCAATGAGCAGCGACT | AGTCGCTGCTCATTGGAGTC |  | 55 | 60.11 | 3 | 2 |
| cDNA_2.1F | GAGTCTTTAAACATGGCTATGAGAA |  | 116 | 36 | 57.15 | 6.0 | 2.00 |
| cDNA_2.1R | CACTCGATGTTCTGCTGCAA | TTGCAGCAGAACATCGAGTG |  | 50 | 58.85 | 5.0 | 3.00 |
| cDNA_2.2F | TAATACCATGAGGGCAGACCC |  | 77 | 52.38 | 58.95 | 4.0 | 3.0 |
| cDNA_2.2R | CTGCTGCAATTGCTTTGTCA | TGACAAAGCAATTGCAGCAG |  | 45 | 57.86 | 8.0 | 3.0 |
| cDNA_3F | CTGACATAGTAGAAGGGGTTGACT |  | 88 | 45.83 | 59.29 | 3.0 | 1.00 |
| cDNA_3R | TCATCTTGGTCCTTTGGGGG | CCCCCAAAGGACCAAGATGA |  | 55.00 | 59.30 | 3.0 | 0.00 |

Table S3: CHD8 cDNA primer sequences.

Note: Primer set 1 and 3 were selected after optimisation as the final two sets for qRT-PCR.


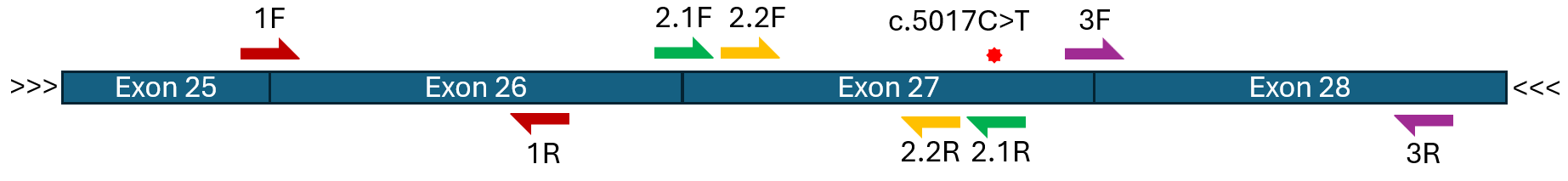
**Figure S2. Location of cDNA primers designed for CHD8 qRT-PCR.** All cDNA primers designed for CHD8 qRT-PCR lies within exon 25 to exon 28. Primer 1F spans across the exon boundary of exon 25 and 26, primer 2.1F spans across the exon boundary of exon 26 and 27, primer 3F spans across the exon boundary of exon 27 and 28. All reverse primers are within exons. Primer 2.1R covers the current variant.

Table S4: Clinical profile of the atypical RTT case per Neul's Revised clinical diagnostic criteria for RTT^1^.

| [A] Rett syndrome (RTT) diagnostic criteria | | | Typical | Atypical | Y/N |
| --- | --- | --- | --- | --- | --- |
| *Required criterion  (typical/atypical RTT)* | 1 | A period of regression followed by recovery or stabilization | Yes | Yes | Y |
| *Main criteria  (Typical RTT)* | 1 | Partial/complete loss of acquired purposeful hand skills | ALL | ≥ 2 | N |
|  | 2 | Partial/complete loss of acquired spoken language |  |  | Y |
|  | 3 | Gait abnormalities: Impaired (dyspraxic) or absence of ability |  |  | Y |
|  | 4 | Stereotypic hand movements such as hand wringing/squeezing, clapping/tapping, mouthing, and washing/rubbing automatisms |  |  | Y |
| *Exclusion criteria  (Typical RTT)* | 1 | Brain injury secondary to trauma (peri- or postnatally), neurometabolic disease, or severe infection that causes neurological problems | NONE | NONE | N |
|  | 2 | Grossly abnormal psychomotor development in first 6 months of life |  |  | N |
| *Supportive criteria  (Atypical RTT)* | 1 | Breathing disturbances when awake | Not necessary | ≥ 5 | Y |
|  | 2 | Bruxism when awake |  |  | Y |
|  | 3 | Impaired sleep pattern |  |  | Y |
|  | 4 | Abnormal muscle tone (hypotonia/hypertonia) |  |  | Y |
|  | 5 | Peripheral vasomotor disturbances |  |  | N |
|  | 6 | Scoliosis/kyphosis |  |  | N |
|  | 7 | Growth retardation |  |  | N |
|  | 8 | Small cold hands and feet |  |  | N |
|  | 9 | Inappropriate laughing/screaming spells |  |  | Y |
|  | 10 | Diminished response to pain |  |  | N |
|  | 11 | Intense eye communication - ‘eye pointing’ |  |  | N |
| [B] Other atypical forms of RTT | | | | | |
| Variant types | | Phenotypes | Molecular genetics | | Y/N |
| *Preserved speech variant (Zappella variant)* | | • Regression at 1-3 years • Mild regression of hand skills, intellectual disability (IQ ≤ 50) • Language recovery; autistic behaviours • Decreased frequency of typical RTT features | Predominantly *MECP2* mutations | | N |
| *Early seizure variant (Hanefeld variant)* | | • Early onset of seizures • Decreased frequency of RTT features | Rarely *MECP2* mutations, *CDKL5* variants possible | | N |
| *Congenital variant (Rolando variant)* | | • Grossly abnormal initial development, early regression <5 months • Postnatal microcephaly <4 months • Lack of typical intense “RTT” eye-gaze • Typical RTT autonomic abnormalities | Rarely *MECP2* mutations, *FOXG1* variants possible | | N |

Notes: Y, Yes; N, No; IQ, Intelligence quotient; *MECP2,* Methyl-CpG-Binding Protein-2; *CDKL5*, Cyclin-dependent Kinase-like 5; *FOXG1*, Forkhead box G1; RTT, Rett Syndrome.
